# Case of Amputation at the Shoulder-Joint

**Published:** 1816-08

**Authors:** J. B. Whitridge


*For the London Medical and Physical Journal.*

### *Case of Amputation at the Shoulder-joint.* Reported by J. B. Whitridge, M. D. of Charleston, S. C.

AT the battle of Stoney Creek, in Upper Canada, on the 6th of June 1813, William Peters, of the 2d regiment of artillery, was wounded in the shoulder. A musket ball of the the largest size, entered just below the origin of the short head of the biceps flexor cubiti muscle; passed in an oblique direction through the head of the os humeri, and made its exit near the attachment of the middle portion of the deltoid muscle, to the acromion process of the scapula. The wound produced very considerable contusion, and unusual laceration, which induced some gentlemen to believe, that the wound was occasioned by a grape or cannister shot. From the deficiency of surgeons, I here acted as surgeon to the first brigade commanded by Brigadier-general John Chandler, under an order from Major-general Dearborn; there being no other medical officer attached to it. The other officers of the medical staff belonging to this brigade were left behind, in charge of the sick at Newark; whilst I was ordered on with this division of the army, upon the expedition against Burlington Heights, at the head of Lake Ontario.

In consequence of the retreat from Stoney Creek, the situation of the wounded was deplorable. They were hastily thrown into waggons, and transported to a place called Forty-mile Creek, a distance of fourteen miles, over the worst waggon road that can possibly be conceived.

At Forty-mile Creek, they were placed in barns during a rainy night, and the next day I received an order from Colonel Burn (then commanding officer of the division, after the capture of Generals Chandler and Winder,) to take all the wounded, both British and American, and transport them in boats to Fort George, and place them in the General Hospital at Newark. This order was executed.

I took them in open boats (operating and dressing wounds while the day-light lasted; and the next day also, when I arrived at Fort George) and on the 8th delivered them to the hospital surgeon at Newark. As he had but few assistants, I remained with them two or three days, after which, I did not see Peters nor any of them again for several weeks. During these few days, his wound was extremely painful.

In the latter part of June, I was requested by the attending surgeon to see him, which I did, and recommended amputation at the shoulder-joint, an operation which at that time might have been performed with much more ease, and with much greater hope of success, than at any subsequent period, though rendered difficult, by the extremely diseased state of the bones, muscles, and integuments, and particularly by the great waste of the soft parts, in consequence of an extensive suppuration.

The attending surgeon, and all those attached to the General Hospital, declined so desperate an operation. The soldier also, at first refused to submit to the operation, in consequence of the faint hope of cure, held out to him by Dr. D. the hospital surgeon, into whose charge he was delivered.

After this I saw him several times, he still persisted in his obstinacy, whilst his constitutional as well as his local disease were both daily becoming very much worse.

Being an extremely timid irresolute man, he suffered his disease to advance (notwithstanding all that was said to him) until the last of July, when he sent for me to operate; being now persuaded, as I before told him, that, without an operation, death was inevitable. On examination I found him extremely emaciated. He was upon the very verge of the grave, being excessively reduced by cough, diarrhœa, hectic fever, and copious suppuration, so that he probably could not survive eight and forty hours.

I at first thought it was too late even to attempt an operation; that the integuments had become so diseased, that one could not be performed; and, if it were, it was believed, that there was not more than one chance out of twenty for his recovery; and that it was very questionable whether the patient would be able to survive the operation. Finding, however, that the soldier had acquired resolution and courage in the last expiring moments of life, I resolved to give him the only chance which my art afforded. "*Anceps remedium potius quam nullum.*"

I devised a plan of operating,* the only one in my judgment which promised the least hope of success; and the next morning called a consultation of all the medical staff at the post. To them I communicated my plan; they acceded to it, and the operation was agreed on.

Every thing being prepared, the patient was seated upright on a table of convenient height, under an harbour, and supported by assistants. The occurrence of hæmorrhage during the operation was guarded against by an assistant who made compression upon the subclavian artery, where it passes over the first rib immediately above the clavicle.

The arm being raised to an angle of about seventy-five degrees from the body, i. e. fifteen degrees below a horizontal line, and supported in that situation by another assistant; with a large scalpel, I commenced my first incision upon the top of the shoulder, behind the articulation of the clavicle with the processus acromion. This incision was continued

* Something in the style of Mr. Charles Bell. See his Operat. Surg. vol. I. p. 268, Amer. Edit.

down down upon the outside of the shoulder, in a zigzag direction, quite into the axilla, dividing only the skin and membrana adiposa. At the same point, I commenced my other incision, and continued it down in the same manner upon the inside of the arm, until it met the angle of the former in the axilla, taking care that the curvatures of the one corresponded with the other.

Within these two crooked incisions, I was enabled to include almost the whole of the diseased portion of integuments, which was the object of their curvatures. Each of these flaps were successively raised, and the fibres of the deltoid muscle cut through; then the long head of the biceps flexor cubiti, and the capsular ligament were divided, and the head of the os humeri dislocated, by the assistant who depressed the arm, and slipped the head of the bone from the socket. I then took the catlin, being the most convenient sized knife that I had, and with one sweep divided the short head of the biceps flexor cubiti, coraco-brachialis, and the whole of the remaining muscles, tendons, nerves, and blood-vessels. I immediately seized the artery with my forceps (the flow of blood, however, was completely commanded by the compression which was made above the clavicle) whilst another assistant placed a ligature upon the artery, by which it was effectually secured. Except the axillary artery, one small muscular artery was the only blood-vessel that required a ligature.

In consequence of caries, I next proceeded to remove the coracoid process of the scapula, which I did by means of Hey's saw, and then in like manner, the anterior and superior portion of the processus acromion; both of which I pared down smooth, to prevent any irritation to the integuments.

I then brought down the flaps, which fit so accurately together, as only to form a line, some part of which was horizontal, some part longitudinal, not unlike an artificer's dove-tailing. These were secured by means of strips of adhesive plaster; the usual dressings applied, and over the whole the spica bandage.

During this operation very little blood was lost, the prevention of which was all important, as the loss of any considerable quantity, in his extremely weak and debilitated state, would have proved fatal.

The gentleman placed at the wrist to examine the pulse, to watch the aspect of the patient, and to administer stimulants, as occasion might require, in the midst of the operation, requested me to stop, and let the patient recover a little, tle, as his pulse* had become imperceptible. I did so. Stimulants were repeated, and he soon recovered, so that I was able to go on with the operation. The night following he passed more comfortably than any night previous for some time. The next day he appeared cheerful and animated; seemed much stronger, and took food with relish. On being asked if he felt as well as before the operation, he exclaimed with as much animation as his feeble system would admit; "Yes, (said he,) and a hundred times better."

His cough, diarrhœa, and hectic fever, soon left him. He acquired strength rapidly, and at the first dressing (which was on the third day after the operation,) could rise up in bed without assistance. Some portion of the lips of the wound united by the first intention, and the remaining portion gently suppurated.

There was a large hole left in the integuments, contiguous to the coracoid process, which could not be obviated by the operation. Granulations gradually formed over the remaining portion of this process, which closed the orifice, and the wound occasioned by the operation nearly cicatrized.

The patient was elated with the hope of recovery, and I myself felt flattered with the prospect of success.

This unfortunate soldier, in whose case I felt very much interested, was reported convalescent for four weeks, by the attending surgeon, after which, in consequence of sudden cold, all his horrible symptoms returned. He was attacked with diarrhœa, more severe than ever; colliquative sweats attended with fever supervened, and he died five weeks after the operation.—*New-England Journal.*

*December* 1815.

